# A Novel Technique for Preparation, Staining, and Visualization of Tissue with Metal Implants and Extraskeletal Calcification Areas

**DOI:** 10.17691/stm2020.12.4.02

**Published:** 2020-08-27

**Authors:** R.A. Mukhamadiyarov, L.A. Bogdanov, S.V. Mishinov, A.G. Kutikhin

**Affiliations:** Senior Researcher, Laboratory for Fundamental Aspects of Atherosclerosis, Department of Experimental and Clinical Cardiology; Research Institute for Complex Issues of Cardiovascular Diseases, 6 Sosnovy Blvd, Kemerovo, 650002, Russia;; Junior Researcher, Laboratory for Fundamental Aspects of Atherosclerosis, Department of Experimental and Clinical Cardiology; Research Institute for Complex Issues of Cardiovascular Diseases, 6 Sosnovy Blvd, Kemerovo, 650002, Russia;; Senior Researcher, Neurosurgeon, Neurosurgery Unit; Novosibirsk Scientific Research Institute of Traumatology and Orthopedics named after Ya.L. Tsivyan of the Ministry of Health of the Russian Federation, 17 Frunze St., Novosibirsk, 630091, Russia; Head of the Laboratory for Fundamental Aspects of Atherosclerosis, Department of Experimental and Clinical Cardiology Research Institute for Complex Issues of Cardiovascular Diseases, 6 Sosnovy Blvd, Kemerovo, 650002, Russia;

**Keywords:** scanning electron microscopy, metal implants, stents, vascular mineralization, vascular calcification, implantation, biocompatibility.

## Abstract

**The aim of the study** was to evaluate the efficacy of a novel technique for preparation, staining, and visualization of tissues containing extra-skeletal mineralization areas, all-metal implants or their prototypes for their subsequent examination using scanning electron microscopy in the backscattered electron mode.

**Materials and Methods.:**

After fixation in 10% formalin (24 h), the biomaterial (a titanium nickelide plate with the surrounding tissues after subcutaneous implantation, patented titanium alloy plates with the surrounding tissues after cranioplasty, primary and secondary calcified atherosclerotic plaques) were fixed with 1% osmium tetroxide (12 h) and then stained with 2% aqueous solution of osmium tetroxide (48 h). The samples were further stained with 2% alcoholic uranyl acetate (5 h), dehydrated with isopropanol (5 h) and acetone (1 h), impregnated with a mixture of acetone and epoxy resin Epon (1:1, 6 h) and then embedded into a fresh portion of epoxy resin (24 h), which was followed by polymerization at 60°C. After grinding and polishing, epoxy blocks were counterstained with lead citrate (7 min) and sputter-coated with carbon, then the samples were visualized by scanning electron microscopy in the backscattered electron mode. The elemental composition was studied using X-ray microanalysis.

**Results.:**

The developed technique allows obtaining high-quality images at five thousand-fold magnifications, provides the possibility to identify the shape and structure of intact metal and mineral inclusions, and to type the surrounding cells, distinguishing mesenchymal and immunocompetent cells by shape and cytoplasmic content. Apart from connective tissue capsule thickness and leukocyte infiltration, this technique makes it possible to estimate the number and area of newly formed small-caliber vessels representing a surrogate marker of inflammation.

**Conclusion.:**

The proposed technique provides the possibility to investigate adequately the structure of samples when their sectioning is impossible or significantly complicated, with image quality remarkably higher than that obtained by light microscopy.

## Introduction

Despite the rapid evolvement of histological and immunohistochemical methods, improvement of imaging techniques, development of new models of light, fluorescence, and electron microscopes [1–3], to date there are no effective methods for histological analysis of tissues containing all-metal implants and ectopic calcifications [4].

Stents [5, 6] and metal plates [7, 8] are the most commonly used metal implants. To investigate biocompatibility of promising metal alloys (for example, titanium nickelide), it is required to implant medical device prototypes made of the above alloys into laboratory animals, which is followed by assessment of connective tissue capsule formation, infiltration by immunocompetent cells and neovascularization [9, 10].

At the same time, when sectioning with a microtome or cryotome, metal structures inevitably separate from the surrounding tissue due to mismatch in tissue density, which causes tissue rupture and disrupts its integrity irreversibly. A similar problem is also observed in the presence of extra-skeletal mineralization areas in the tissues, which is characteristic, for example, for atherosclerosis [11], medial vascular calcification [12], or calcification of native [13] and prosthetic [14] heart valves.

On the other hand, routine histological or immunohistochemical staining requires visualization by light microscopy, the limited resolution of which does not allow obtaining high-quality images at more than 400-fold magnification. It is unlikely to solve this problem even with confocal microscopy that, firstly, allows no adequate assessment of tissue structure and, secondly, is usually limited by 630-fold magnification. With this range of magnifications, it is rather difficult to carry out adequate assessment of neovascularization, which is a surrogate marker of inflammation during the implantation of medical device prototypes. Besides, in practice, newly formed small-caliber vessels are rarely stained with antibodies to classical endothelial markers.

Therefore, it is necessary to come up with a fundamentally new technique for preparation, staining, and visualization of tissues containing metal implants or ectopic mineralization areas. Earlier, our group developed an original method for prolonged osmium plating of formalin-fixed tissues with their subsequent dehydration, staining with uranyl acetate, embedding in epoxy resin, grinding, polishing, and staining with lead citrate for further investigation using scanning electron microscopy in the backscattered electron mode [15]. The proposed method allows obtaining images similar to transmission electron microscopy, but with lower resolution (however, sufficient for adequate histological analysis) due to the use of dyes for transmission electron microscopy and observance of visualization principles for scanning electron microscopy in the backscattered electron mode [15].

**The aim of the study** was to evaluate the efficacy of a novel technique for preparation, staining, and visualization of tissues containing extra-skeletal mineralization areas, all-metal implants or their prototypes for their subsequent examination using scanning electron microscopy in the backscattered electron mode.

## Materials and Methods

The following objects were taken as reference:

1. a titanium nickelide plate (10×10×1  mm) with surrounding tissues, extracted 2 months after subcutaneous implantation to a Wistar rat;
2. original patented titanium alloy perforated plates, extracted 1 and 2 months after orthotopic implantation to New Zealand white rabbits during cranioplasty;
3. a carotid atherosclerotic plaque with a small amount of intact internal carotid artery tissue obtained from a 60-year-old male during endarterectomy due to hemodynamically significant chronic cerebral ischemia;
4. a fragment of a stented internal carotid artery explanted from a 57-year-old male as a result of repeated carotid endarterectomy due to restenosis, which also caused hemodynamically significant chronic cerebral ischemia.

The study was carried out in accordance with the Rules of Good Laboratory Practice (Russia, 2016), the guidelines of the Declaration of Helsinki (2013), and in compliance with the ethical principles established by the European Convention for the Protection of Vertebrate Animals used for Experimental and Other Scientific Purposes (Strasbourg, 2006). The study protocol was approved by the local ethics committee of the Research Institute for Complex Issues of Cardiovascular Diseases. Written informed consent was obtained from every patient prior to enrollment.

On extraction, tissue samples were placed in buffered (pH 7.4) 10% aqueous formalin solution (BioVitrum, Russia). After 24-hour fixation in formalin (formalin solution being changed after 12 h), the biomaterial was post-fixed with 1% osmium tetroxide in 0.1 M phosphate buffer for 12 h, then stained with 2% osmium tetroxide in bidistilled water for 48 h. The samples were dehydrated with a graded set of alcohol solutions of increasing concentrations (50, 60, 70, 80, and 95% ethanol) in two procedures, each taking 15 min. Next, they were stained with 2% uranyl acetate (Electron Microscopy Sciences, Switzerland) in 95% ethanol (5 h), dehydrated with 99.7% isopropanol (BioVitrum) for 5 h and acetone (Reachim, Russia) for 1 h, impregnated with a mixture of acetone and Epon epoxy (Electron Microscopy Sciences) in 1:1 ratio (6 h). Then the samples were embedded into a fresh portion of epoxy resin (for 24 h), which was followed by polymerization in FixiForm containers (Electron Microscopy Sciences) at 60°С. After that, the samples in epoxy blocks were ground and polished with the TegraPol-11 machine (Struers, Denmark). Staining with lead citrate was carried out during 7 min according to Reynolds by applying the solution to the surface of the polished sample and then washing it with bidistilled water. Next, the polished surfaces of epoxy blocks were sputter coated with carbon (coating thickness 10–15 nm) using a vacuum coating system (EM ACE200; Leica Biosystems, Germany). The structure of the samples was visualized by scanning electron microscopy in the backscattered electron mode using the S-3400N electron microscope (Hitachi, Japan) in the BSECOMP mode at an accelerating voltage of 10 kV.

The elemental composition of the studied samples was investigated by means of X-ray microanalysis using the XFlash 4010 energy dispersive spectrometer (Bruker Daltonics, Germany) as part of the S-3400N scanning electron microscope (Hitachi). Elemental analysis was carried out under low vacuum (pressure in the microscope chamber 20 Pa) and at an accelerating voltage of 15 kV by scanning electron microscopy in the backscattered electron mode without using standard samples.

Digital microphotographs were examined to reveal the location of electron-dense inclusions (metal structures and mineralization areas), evaluate the structural features of biological tissues of the studied samples, identify endothelial cells, mesenchymal cells (smooth muscle cells, fibroblasts), and immunocompetent cells.

## Results

**Figure 1:**
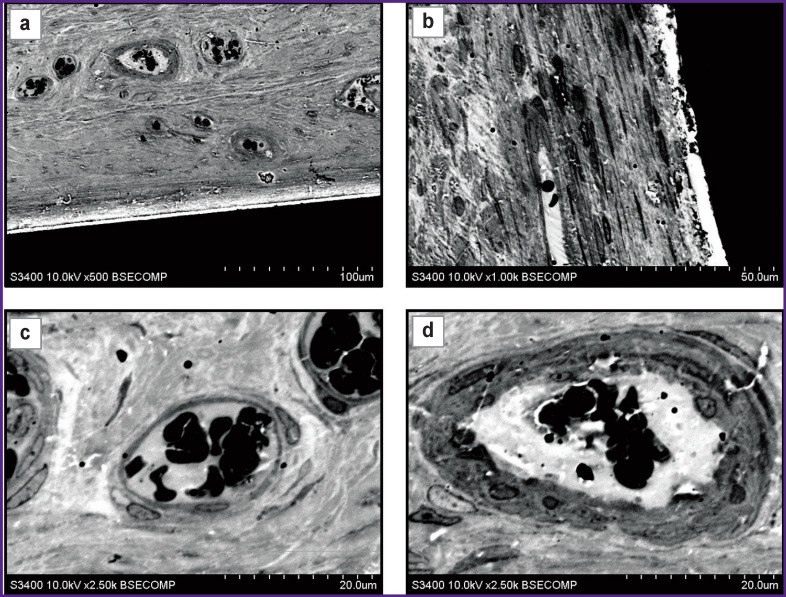
A titanium nickelide plate removed 2 months after subcutaneous implantation to a Wistar rat Visualization of the barrier between the plate and tissues ((a) ×500; (b) ×1000) and newly formed vessels ((c), (d) ×2500)

The proposed method may be widely used to study the interaction of laboratory animal tissues with metal implants, in particular, on the classical model of subcutaneous implantation of medical device prototypes. For example, it is almost impossible to obtain sections of the extracted samples with surrounding tissues due to substantial thickness of titanium nickelide plates (1 mm). Consistent use of grinding and polishing makes it possible to achieve high preservation of biological tissues without damaging the implant (Figure 1 (a), (b)). The fine structures of the sample are visualized in more detail with the increased magnification. In particular, 1000-fold magnification (Figure 1 (c)) makes it possible to evaluate the structural features of tissues and the structure of cells adjacent to metal. The presence of a small slit-like space containing small electron-dense granules was found immediately at the site of contact. Formation of the slit and granules was likely to be an artifact associated with the processes of harvesting and preparing the material. Further, a layer of dense connective tissue represented by fibrocytes with a typical structure of nuclei and cytoplasm was identified reliably in the backward direction from the implant. A characteristic feature of this portion of the sample was the absence of immunocompetent recipient cells. By the time of sampling, the material showed complete biocompatibility.

Abundant loose connective tissue was observed behind the layer of dense connective tissue, and the boundary between them was distinct. It should be noted that large blood vessels were present in the layer of loose connective tissue, in contrast to dense connective tissue (see Figure 1 (c)). As a rule, the maximum number of vessels was detected near the site of contact between loose and dense connective tissues, but the vascular density got lower with the increasing distance from the implant. Large blood vessels had a typical structure (Figure 1 (d)) and showed differences in the structure of the membranes. The endothelial layer was observed in all vessels, though, in some of them there followed several layers of rounded cells with a cytoplasm having increased electron density. Further increase in magnification allows investigating the structure of the cytoplasm and nuclei in detail, making it possible to increase reliability of morphological identification of cells.

**Figure 2:**
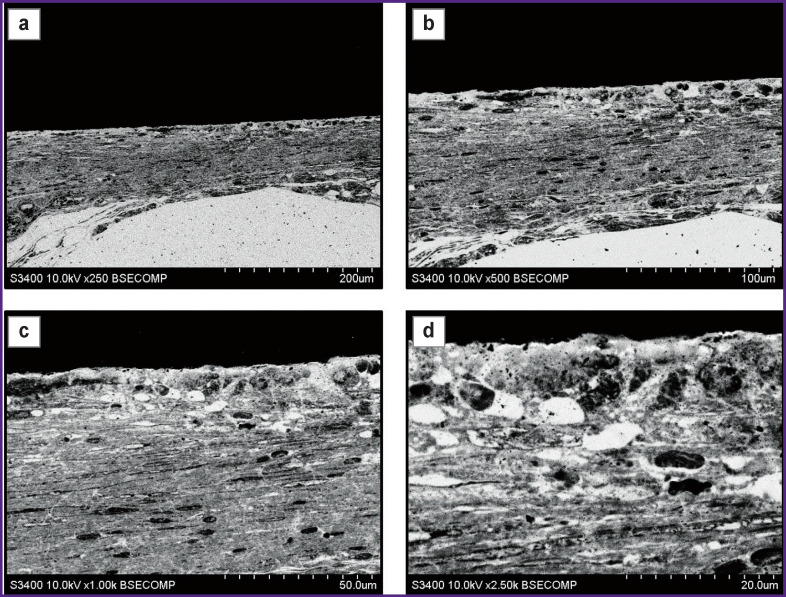
Perforated plate made of patented original titanium alloy, removed 2 months after intracranial implantation to New Zealand white rabbits Visualization of the barrier between the plate and tissues ((a) ×250; (b) ×500) and differentiation of connective tissue into two layers: (c) underlying dense layer containing elongated cells with oblong nuclei, ×1000; (d) the layer adjacent to the plate, loose with rounded intercellular spaces, ×2500

A similar result was observed while examining the explanted titanium alloy mini-plates used as cranial patches in experiments on laboratory rats. Two months after cranioplasty, it was observed that there was a connective tissue layer formed around the structural elements of the plates. At low magnifications (Figure 2 (a), (b)), there was detected a connective tissue layer formed by elongated cells parallel to the surface of the plate. At high magnifications (Figure 2 (c), (d)), two layers were distinguished in the composition of the tissue. In the layer immediately adjacent to the plate, there was a loose arrangement of cells with large transparent rounded intercellular spaces, and following it there was a layer of elongated cells with oblong nuclei and moderate density cytoplasm. Transition between the layers was gradual. In the examined sample area, there was firm adherence of cells to the patch material. Adjacent cells had various shapes (predominantly rounded) and contained small granules in the cytoplasm.

**Figure 3:**
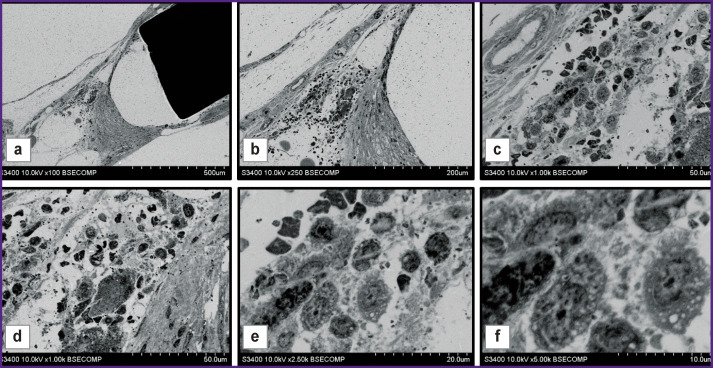
Perforated plate made of patented original titanium alloy, removed 1 month after intracranial implantation to New Zealand white rabbits Connective tissue capsule forming around the plate: (a) general view of the plate with surrounding tissues, ×100; blood vessels and multiple cells of the surrounding tissue: (b) ×250; (c) ×1000; numerous macrophages in the focus of productive inflammation: (d) ×1000; (e) ×2500; (f) ×5000

During the presented experiments, we studied the interaction of cranial patches with the surrounding tissues at the early stages of implantation. The patch sheath was forming unevenly: a layer of fibrous tissue formed along the long patch surface, while the end part of the surface remained clear. There was vast clear space in this region, which contained no structural elements and was surrounded by a thin layer of connective tissue connected to a tissue array that was spaced from the end surface (Figure 3 (a)). Blood vessels and various types of cells (Figure 3 (b), (c)), especially macrophages (Figure 3 (d)–(f)), were present in this tissue. Macrophages had a typical structure and contained vacuoles with transparent content (see Figure 3 (e), (f)). The presence of macrophages is likely to indicate the presence of productive inflammation caused by implantation of artificial biomaterial.

**Figure 4:**
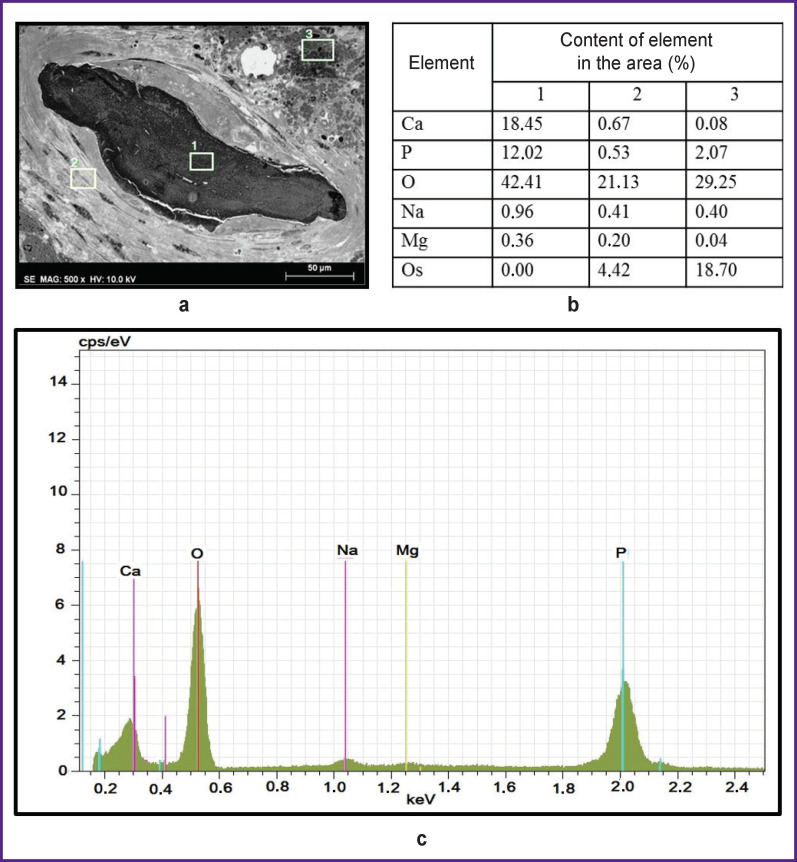
Structure, cellular environment, and chemical composition of the mineralization area as part of atherosclerotic plaques: (a) calcification with the surrounding tissues, ×500; white rectangles with numbers (*1*, *2*, *3*) indicate the areas where the elemental composition was identified (segment *1* — calcification itself; segment *2* — dense connective tissue adjacent to calcification; segment *3* — loose connective tissue abundant in phospholipids); (b) elemental composition of the areas marked in the micrograph; (c) a diagram of X-ray microanalysis of segment *1* (calcification)

Investigation of histological material containing large mineral inclusions also presents serious methodological difficulties. The areas of mineralization have high hardness, mechanical strength, and are often heterogeneous, which prevents high-quality sectioning. Grinding and polishing instead of sectioning are likely to solve this problem. Figure 4 shows the results of examining the mineralized site of an atherosclerotic plaque. The mineral deposits visualized in this area were large electron-dense inclusions inside the neointima with an inhomogeneous structure (Figure 4 (a)). The calcification area was surrounded by a layer of weakly-structured vacuolated material with a moderate electron density. Next to it, there were several layers of smooth muscle cells.

Another important advantage of the proposed method is the ability to carry out elemental analysis to determine the phase of calcium phosphate or another mineral, which is important for assessing the mineral maturity, the calcification site age, and can affect the stability of the atherosclerotic plaque. Evaluation of elemental analysis results for the three sites (Figure 4 (b), (c)) showed that calcium, phosphorus, oxygen, sodium, magnesium, and osmium were present in them.

Oxygen, calcium, and phosphorus prevailed in the mineralization area (segment *1*), which indicates its phosphate-calcium nature. It should be noted that osmium used for post-fixation of tissue caused no artifact contamination of calcium inclusions and, consequently, biased results of elemental analysis.

In the adjacent connective tissue calcification site (segment *2*), the amount of calcium and phosphorus was about 0.5%, which is a high level for biological tissue and it may reflect active metabolism of these elements in smooth muscle cells. In addition, certain osmium content was detected in this segment (about 4%).

In loose connective tissue (segment *3*) with a high electron density, the calcium content was 8 times lower than in the connective tissue adjacent to the mineralization area, but the amount of phosphorus and osmium was 4 times higher. The high content of these elements suggests that in this case, the high electron density of the area results from presence of a large number of phospholipids characteristic of atherosclerotic plaques, since they are highly osmiophilic and contain phosphorus, but not calcium.

**Figure 5:**
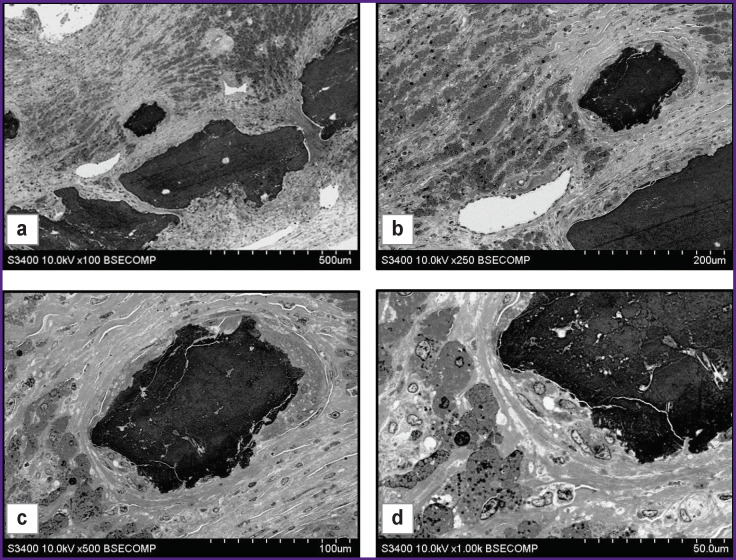
Mineralization areas with surrounding tissues as part of atherosclerotic plaques: (а) general view of calcifications and their location in the plaque structure, ×100; (b) location of a newly formed large blood vessel close to calcification, ×250; (c) connective tissue capsule around calcification, ×500; (d) multiple cells of various structures close to calcification, ×1000

Thus, the proposed method has shown its efficacy in the investigation of biological samples with mineral inclusions, making it possible to evaluate not only the morphological characteristics, but also the elemental composition of inclusions and the surrounding tissues. In cases where there is no need to study the elemental composition of samples, mineral inclusions can be identified based entirely on morphological features. The general structure of the object with the surrounding tissues and location of blood vessels are clearly visible when studied at low magnifications (Figure 5 (a), (b)). Higher magnification (Figure 5 (c), (d)) makes the surroundings of the calcification area, its heterogeneous structure, its connective tissue capsule, and the structure of the surrounding cells visible in detail.

**Figure 6:**
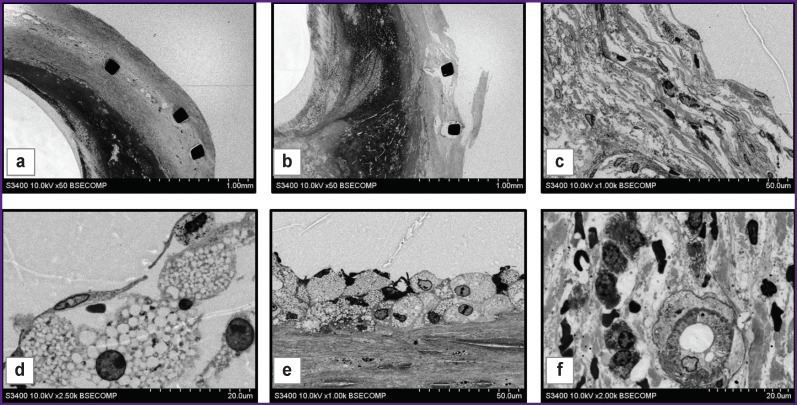
The structure of an atherosclerotic plaque formed in the carotid artery after stenting and restenosis development: (a), (b) general view of the structure of the removed plaque containing the stent and mineralization areas, ×50; (c) partially disintegrated dense connective tissue with immunocompetent and connective-tissue cells, ×1000; (d) intact endothelial cells and foam cells located beneath them, ×2500; (e) a layer of foam cells and underlying dense connective tissue with slit-like spaces, ×1000; (f) a newly formed blood vessel in neointima with multiple cells located around, ×2000

The use of this method for examination of samples containing both metallic and mineral inclusions is of particular interest. In fact, atherosclerotic plaques that developed after stenting, have signs of restenosis and contain mineral inclusions (Figure 6 (a), (b)) can serve as an example. Careful sampling of the material ensured high preservation of the plaque layers. Supports of the stent surrounded by a layer of dense connective tissue were visualized clearly. In the direction peripheral to the vessel lumen, there was observed a layer of dense connective tissue, which was the remaining vessel media present in the vessel structure prior to stenting (see Figure 6 (a)). Between the stent elements, large blood vessels with thin walls were observed. Deeper than the stent, in the direction of the vessel lumen, there was a thick layer of neointima with a different electron density in the radial direction (see Figure 6 (b)). The above figures show areas with high electron density, which can be identified as calcified areas based on morphological features.

At higher magnifications, the cells present in the structure of the samples were well visualized. In particular, Figures 6 (c) and (d) show two different plaque portions in direct contact with the bloodstream and with intact endothelial layers. Endothelial cells had similar structure, but the underlying cells and tissues were different. Figure 6 (c) shows the segment where there was observed a layer of connective tissue under the endothelium, consisting of dense fibers between which there are cells described as smooth muscle cells and macrophages, based on morphological characteristics, these macrophages containing small light vacuoles and lysosomes. It is rather obvious that gradual destruction of dense connective tissue structure occurred in this area. In the variant presented in Figure 6 (d), loose tissue with large intercellular spaces was found under the endothelial layer. The cells adjacent to the endothelium had a rounded shape, round nuclei with a thin layer of chromatin adjacent to the nuclear membrane and diffuse arrangement in the central part of the nucleus. These cells were morphologically similar to foam cells.

In the direction peripheral to the vessel, the layer of foam cells was adjacent to partially preserved layer of dense connective tissue where cells and slit-like spaces were observed (Figure 6 (e)). The inner part of the neointima was heterogeneous. There were no cellular structures in one part of it, while newly formed blood vessels (the so-called vasa plaquorum), macrophages, smooth muscle cells were observed in other parts (Figure 6 (f)).

## Discussion

The original preparation method developed in this work provides an adequate solution to the problem of investigating samples with structural elements that impede sectioning (for example, metal or mineral components). The proposed method has two fundamental aspects: replacement of sectioning procedure by grinding after embedding the sample in epoxy resin, and visualizing its structure by scanning electron microscopy in the backscattered electron mode instead of transmission electron microscopy. Moreover, the images have almost complete visual similarity to those obtained using transmission electron microscopy.

Despite certain difficulty in understanding the key point of the process, the method itself is quite simple and easy to reproduce. To use it, no special modifications of equipment are required; it is possible to achieve high image quality with serial devices (standard models of scanning electron microscopes). It allows obtaining a better high-contrast image through the procedures of prolonged osmosis, residence in alcohol solution of uranyl acetate, and contrasting with lead citrate.

Impregnation of the studied objects with low viscosity epoxy resins (Epon, Spurr resin) makes it possible to investigate large images. In particular, the samples used in the present work were more than 5–7 mm thick. Under this condition, the length and width of the sample have no crucial significance. It should be noted that the linear dimensions of the sample do not affect the quality of grinding and polishing.

The above features of the proposed method provide the opportunity for detailed investigation of biological structures with massive hard inclusions that prevent sectioning. It allows studying not only the surrounding tissue structure, but also the structure of inclusions. This is necessary when the nature of objects is unknown or they have been specifically obtained as implants using original methods (for example, sintering or 3D printing) and their structure determines interaction with biological tissues.

The above results show that analysis of the obtained images allows adequate identification of cells proper and some intracellular structures, in particular, nuclei, cytoplasm, and vacuole. Based on these characteristics, the shape and localization of cells, it is possible to identify not only their type, but also the tissues they form and their physiological state.

The disadvantage of the method is a relatively low resolution: high-quality images can be obtained at the maximum magnification of five thousand times, which is substantially lower as compared to the capabilities of transmission electron microscopes of a similar class. This is attributable to the fact that the image quality depends on the depth of the layer into which the electrons penetrate. When reflected from deep layers, the trajectory of backscattered electrons deviates, which leads to blurring of the image. Possible solutions to this problem may be improvement of staining techniques and optimization of visualization modes.

## Conclusion

The presented original method for preparation, staining, and electron-microscopic visualization provides the possibility to investigate adequately the structure of samples when sectioning is impossible or significantly complicated. However, image quality is remarkably higher than that obtained by light microscopy.
